# Neural stem cell fate: a tango of mechanics and genetics

**DOI:** 10.1038/s44319-026-00718-3

**Published:** 2026-02-17

**Authors:** Mireia Pampols-Perez, Víctor Borrell

**Affiliations:** Instituto de Neurociencias, Consejo Superior de Investigaciones Científicas & Universidad Miguel Hernández, Sant Joan d’Alacant, 03550 Spain

**Keywords:** Neuroscience, Stem Cells & Regenerative Medicine

## Abstract

A new study sheds light on the functional interplay between mechanical forces and molecular signals during early brain development.

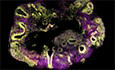

## Teaser

Our body is shaped during development under the control of genetic programs, but also under the influence of mechanical forces. Tissue growth and form is limited, biased and even instructed by mechanical forces. Brain development is also under the influence of mechanical forces, but the extent of their effect, and the interplay with genetic programs, remain fundamentally unknown. New research in *EMBO Reports* (Lampersperger et al, 2026) demonstrates that mechanical forces acting on human neural stem cells (NSCs) bias their cell fate, supporting a key role for mechanics in brain morphogenesis.

Mechanical forces act on growing tissues, shaping their intricate architecture in profound ways during development. This has been deeply studied and is well understood in some specific contexts like heart looping (Voronov et al, 2004), cardiovascular development (Pampols-Perez et al, 2025), and gut homeostasis (Baghdadi et al, 2024). In contrast, brain development is a process mostly recognized as being governed by the combination of genetic instructions and molecular signaling pathways, where the involvement of mechanical forces is much less clear in spite of scattered evidence at macroscopic and microscopic levels (Thompson et al, 2019; Franze, 2013). Winds of change begin to wonder about how mechanics and molecular mechanisms may integrate and influence each other while regulating brain development, questions that remain largely uncharted territory. New work published now in *EMBO Reports* (Lampersperger et al, 2026) delves deeply into this frontier, demonstrating that variations in the mechanical conditions of human brain organoid culture can bias NSCs toward distinct fates. This groundbreaking work uncovers a vital regulatory link between tissue mechanics and lineage decisions during early neurodevelopment via changes in gene expression, highlighting how previously underestimated forces play a starring role alongside molecular signals.

NSCs are the core architects of the brain, producing during development, at the right time and in the correct proportions, the broad range of neurons and glial cell populations that ultimately assemble into regionally specialized brain structures (Taverna et al, 2014). Models of this intricate process have classically focused on the existence and effects of several factors: morphogen gradients, patterns of molecules that provide spatial cues; transcription factors, DNA-binding proteins that orchestrate the unfolding of genetic programs; and temporal patterns, which control the timing of developmental events with Swiss precision. Unfortunately, the role of mechanical forces in early brain development has remained largely unexplored, as analyzing their effects on neural stem cell biology and fate in living tissue has proven particularly challenging, and isolating physical from chemical effects poses significant experimental challenges. Studies across a wide range of developmental systems have hinted that biomechanical properties such as tissue stiffness, cytoskeletal tension, and mechanical stresses can guide stem cell trajectories (Heisenberg and Bellaïche, 2013). This body of evidence raises the intriguing possibility that a sophisticated “mechanical code” may operate in parallel with chemical cues, regulating brain development every step of the way.

**Figure 1:**
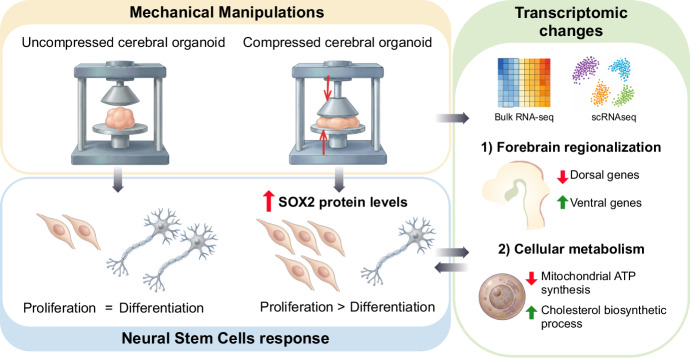
Mechanical compression directs the fate of neural stem cells. In cerebral organoids, mechanical compression increases SOX2 protein expression in neural stem cells, redirecting their fate from neuronal differentiation to self-renewal, which consequently diminishes neurogenesis. Mechanical compression also affects the gene regulatory networks that control tissue patterning and cellular metabolism, related to SOX2 transcriptional control. Illustrae was used to generate some elements composing this figure.

To explore this fundamental question with the required rigor, Lampersperger et al came up with a clever approach, turning to cerebral organoids (COs) derived from human induced pluripotent stem cells. COs are a controlled dish-based approach that recapitulates many aspects of early embryonic brain organization in vivo, thus serving as a viable proxy of the early human brain primordium that is experimentally accessible. By carefully imposing mechanical perturbations such as acute short compressions, or persistent alterations of the organoid microenvironment such as changes in substrate stiffness or spatial confinement, the researchers reveal that tissue mechanics has a significant effect on NSC lineage decisions (Fig. 1). Mechanical forces shift the balance between proliferation and differentiation/neurogenesis of NSCs, via regulating the levels of SOX2 protein, a transcription factor key for NSC self-renewal. Upon compression of NSC-filled organoids, their levels of SOX2 are upregulated, and NSCs switch their fate, from producing neurons to generating more NSCs, thus decreasing neurogenesis. To elucidate the molecular mechanisms underlying these effects, the authors elegantly exploited deep sequencing approaches, combining bulk RNA profiling and single-cell transcriptomics. Their analyses revealed the downstream consequences of their mechanical manipulations: entire gene expression networks had been reprogrammed, now linked to early patterning events and cell-type-specific metabolic pathways. In essence, the results of Lampersperger and colleagues demonstrate that mechanical forces are not passive bystanders during early stages of brain development, but rather exert a very strong influence. Rather than simply reflecting the outcomes of growth with no other consequence, forces impinging on early neural tissue are actively translated into distinct transcriptional states that dictate whether NSCs maintain their progenitor identity or embark on taking neuronal or glial paths.

The new study by Lampersperger and colleagues is a cornerstone in the compelling argument that the consideration of mechanics in neurodevelopmental control must be elevated from a merely permissive backdrop to a fully instructive component, on par with genetic and biochemical regulators. The strategic use of human COs proves beautifully invaluable here, enabling precise, titratable mechanical perturbations coupled with comprehensive molecular readouts that capture both population-level trends and single-cell heterogeneity. Nevertheless, as with any organoid-based research, familiar caveats must be acknowledged: these “miniature” brains bear some resemblance to the early telencephalic primordium but are far from fully replicating the cellular complexity and three-dimensional architecture, vascularization, and immune interactions of the brain in a human embryo. In addition, the magnitude, spatial distribution, and timescale of the applied forces in the dish may diverge significantly from the dynamic and pulsatile mechanical landscapes encountered during natural gestation, where heartbeats, maternal movements, and tissue expansions generate a symphony of stresses.

It is critical to note that while the Lampersperger study demonstrates the phenotypic and transcriptomic outcomes of mechanical modulation, the specific mechanosensor molecules that translate these extracellular forces into intracellular transcriptional changes remain unidentified in this neural context. Promising candidates beckon for future scrutiny, such as the mechanosensitive ion channel Piezo1, which has been implicated in toggling NSC fates between neurogenesis and astrogenesis via downstream YAP/TAZ signaling (Pathak et al, 2014) - a pathway exquisitely sensitive to cytoskeletal forces. Along this line, a study in mice showed that removal of Piezo1 leads to defects in cortical development, including altered cortical thickness, consistent with impaired control of progenitor proliferation and differentiation (Nourse et al, 2022). In-depth studies of these molecular intermediaries may unlock the precise wiring diagram of mechanotransduction in the early embryonic brain, particularly neurogenesis and fate of NSCs. Looking ahead, the most impactful extensions of this work will come when organoid insights are bridged back to in vivo mechanical landscapes. Advanced imaging of force distributions in mouse or human embryos, meticulous mapping of Piezo-dependent signaling cascades, and probing the effects of mechanosensing disruption on brain development will bring the field to the next level of understanding the occurrence of neurodevelopmental disorders characterized by aberrant tissue architecture, like lissencephaly and microcephaly.

The remarkable study by Lampersperger and colleagues introduces the paradigm-shifting notion that physical forces actively bias NSC lineage decisions (so far attributed to genetic mechanisms), a new and transformative concept. Brain organoid engineering is now up to a revolution by implementing tissue mechanics into the equation, with the promise of producing more physiologically faithful organoid models. Incorporating tunable mechanical niches will enhance their faithfulness for disease modeling, drug screening, and basic research. Lampersperger et al have only scratched the surface of the tango danced between mechanical forces and genetic mechanisms, inviting the developmental neuroscience field to listen more attentively to the subtle forces whispering within the growing brain.
